# Nano sand filter with functionalized nanoparticles embedded in anodic aluminum oxide templates

**DOI:** 10.1038/srep37673

**Published:** 2016-11-23

**Authors:** NguyenThi Phuong, Anugrah Andisetiawan, Do Van Lam, Jeong Hwan Kim, Doo-Sun Choi, Kyung-Hyun Whang, Jeasun Nham, Yun Jung Lee, Yeong-Eun Yoo, Jae Sung Yoon

**Affiliations:** 1 Nano Mechanical Systems Research Division, Korea Institute of Machinery and Materials (KIMM), 156 Gajeongbuk-ro, Yuseong-gu, Daejeon 34103, Korea; 2 Department of Nano Mechatronics, Korea University of Science and Technology (UST), 217 Gajeong-ro, Yuseong-gu, Daejeon 34113, Korea; 3 Department of Energy Engineering, Hanyang University, 222 Wangsimi-ro, Seongdong-gu, Seoul 04763, Korea

## Abstract

Since the ancient Egyptians had used sand as filter media for water purification, its principle has been inherited through generations and it is still being used now in industries. The sand filter consists of sand literally, and the voids within the sand bed are the pores for filtration. Here we present a filtration principle using nanoparticles, so that the voids between the nanoparticles can be considered as effective pores in nanoscale dimension. Anodic aluminum oxide (AAO) membrane has been used as the working template, and the nanoparticles have been injected and embedded within the pores of the AAO template. Nanoparticles with multiple sizes have been used in order to obtain smaller voids. Moreover, the nanoparticles have been functionalized, or electrically charged, with arginine/phenylalanine (RF) peptide group. In this way, filtration performance for charged particles or molecules, such as methylene blue, has been enhanced. Consequently, this study is expected to provide a new principle for fabrication of nano voids, or nano pores, and for filtration in nanoscale dimension.

Clean water has always been critical for human life and it is becoming more important nowadays because a lot of modern industries require clean water in their manufacturing processes, such as food, medicine, energy, semiconductor and so on. Thus, increasing demand for clean water has brought about various studies on water purification, which may be categorized into disinfection, decontamination, reuse and desalination^1^. Currently, polymeric membrane technology is widely used for filtration and desalination of water. Various polymeric membranes have been developed for different applications, such as microfiltration (MF), ultrafiltration (UF), nanofiltration (NF), reverse osmosis (RO) and membrane distillation (MD)^2^,^3^,^4^,^5^. Recent progress of nanofabrication techniques has enabled diversity of materials and pore geometries for membranes. Accordingly, its applications have been extended to separation of biomolecules, biosensing, DNA sequencing and drug delivery, as well as water purification^6^,^7^,^8^. Meanwhile, sand bed has been used as filtration medium for a long time in history and its principle is still utilized for water purification industry. With a lot of advantages, the sand filter is known to be a simple and cost effective method for filtration or pretreatment of waste water^9^,^10^,^11^,^12^. Effective size of filtration in the sand filter depends on the size and geometry of the filter media, which are usually quartz sand, silica sand, fly ash, furnace slag and so on. If the size of the sand is smaller, finer filtration could be expected. So there have been attempts in order to replace the sand with smaller particles. Nanoparticle may be a good candidate for this purpose because its fabrication has been well established and various applications are expected also^13^,^14^. Therefore, several researches have been conducted on nanoparticle-coated membranes, where voids between the nanoparticles can be utilized as filtration channels or effective pores^15^,^16^,^17^,^18^,^19^,^20^. This principle has several technical issues and one of them is the stabilization of the nanoparticles. Stabilization is required in order to fix the nanoparticles each other or to fix the particle layer on the support layer. Polymer nanoparticles can be crosslinked^17^,^18^ or heated^19^,^20^ to coalesce each other, resulting in nano-porous layer. Another issue is the crack of the particle layer^15^,^16^, which may be a defect on the separation performance. Arrangement of the nanoparticles is another issue because they need to be well-ordered in crystalline arrays to build coalescence between the nanoparticles. If filtration, or separation, is based on size selection principle only, it will be accompanied with high flow resistance. Another principle, such as electric charge selection^16^,^19^,^20^, may provide comparable filtration performance with less flow resistance. Peptide of arginine (R) and phenylalanine (F) is known to be electrically charged, by which nanofibers could be fabricated and self-assembled^21^,^22^. This study introduces a nano sand filter, where nanoparticles are embedded in a porous template so that voids between nanoparticles can be considered as effective pores in nanoscale dimension. By this way, elaborations to prevent crack, or to enhance arrangement and coalescence of the nanoparticles are not required. Nanoparticles with different size have been embedded in order to reduce the size of the voids. And the surfaces of nanoparticles have been functionalized to have arginine/phenylalanine (RF) peptide group, which has electric charge, in order to improve filtration performance for charged particles or molecules.

## Results

### Fabrication: Embedding nanoparticles within pores of AAO templates

**Figure 1:**
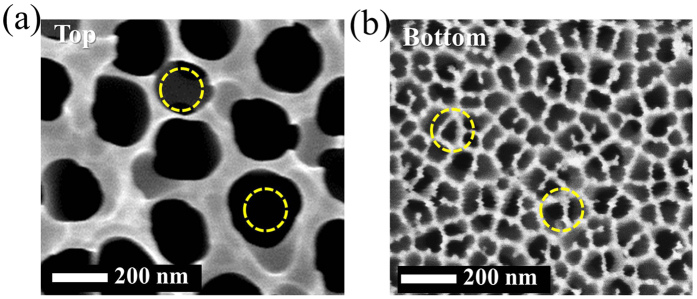
Pore size comparison of working template (AAO). **(a)** Top side. **(b)** Bottom side. Circles represent spherical nanoparticles with diameter of 150 nm.

**Figure 2:**
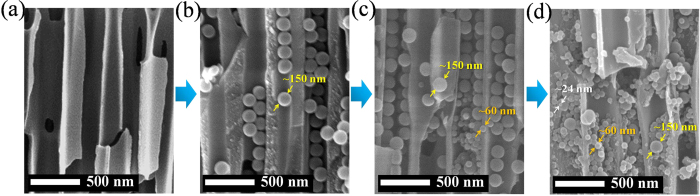
Cross sectional view of working templates for every step of embedding nanoparticles. **(a)** Original AAO template. **(b)** After embedding with 150 nm particles. **(c)** After embedding with 150 and 60 nm particles. **(d)** After embedding with 150, 60 and 24 nm particles.

**Figure 3:**
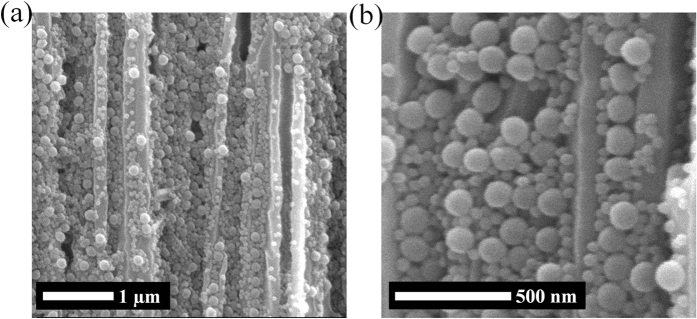
Cross sectional view of AAO template with nanoparticles of 150, 60 and 24 nm. The template was frozen and cut so that embedment of particles could be observed. Magnification of **(a)** 50 000 and **(b)** 150 000.

We used commercial anodic aluminum oxide (AAO) membrane filters (Anodisc, Whatman plc.) as working templates, which have nominal pore diameter of 100 nm. This AAO template has asymmetric pores as seen in Fig. 1, where the pores on one side are bigger than those on the other side. And circles are also illustrated together in Fig. 1, which represent spherical particles with diameter of 150 nm. So it is expected that the particles with this size can be injected into the larger pores but they will be trapped, or embedded, inside. Therefore, solution of nanoparticles of 150 nm in diameter was injected firstly with a syringe pump. Then solutions with particles of 60 and 24 nm were injected in sequence. Figure 2 shows cross sectional scanning electron microscopy (SEM) images for every step. However, it needs to be mentioned that these images may not represent the exact arrangement of the nanoparticles in the AAO pores, because many of them have been lost when the AAO template is cut. Though, we could check whether the particles of every size were embedded within the AAO templates or not. Initially, the pores of original AAO template (Fig. 2a) is filled with 150 nm particles (Fig. 2b), and the following images (Fig. 2c and d) show that the smaller particles are occupying the pores of AAO template together. For further investigation on the embedment of the particles in the pore, the particles are required to remain on the cross section as many as possible, when the template is cut. So after the embedment, the template with nanoparticles of 150, 60 and 24 nm was frozen and then cut. By this way, another cross sectional images were obtained (Fig. 3), which should be more close to actual embedment of the particles in the pores. Meanwhile, all the pores need to be filled with the nanoparticles because this template is for filtration or separation. If there exist any empty pores on AAO template, they will be serious defects for the performance. In this study, the particles are transported in liquid solution, which tends to flow toward unoccupied pores naturally. Therefore, it can be expected that all the pores contain nanoparticles inside, without any empty pores. And overall view of the cross section of AAO template is shown in Supplementary Fig. 1. This shows internal structure of the template together with the nanoparticles in the pores.

### Surface functionalization of nanoparticles with arginine/phenylalanine peptide ((RF)_4_)

**Figure 4:**
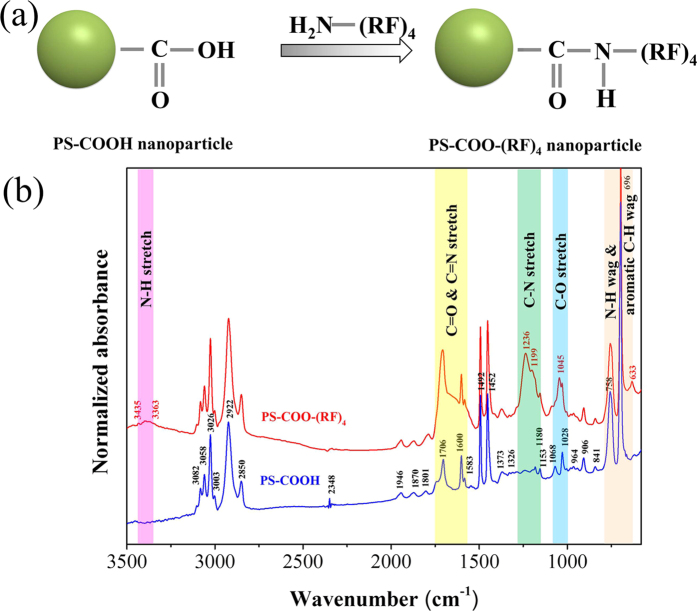
Surface functionalization of nanoparticles. **(a)** Schematics of arginine/phenylalanine peptide ((RF)_4_) functionalized on carboxylated polystyrene nanoparticle (PS-COOH). **(b)** FTIR (Fourier transform infrared spectroscopy) analysis of nanoparticles.

**Figure 5:**
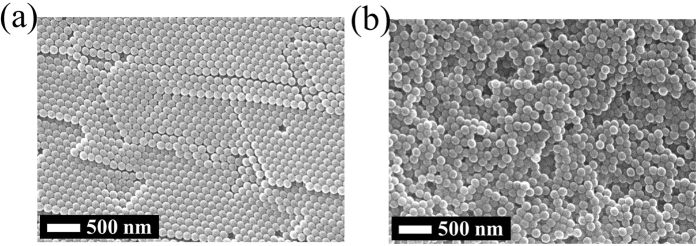
Comparison of the arrangement of nanoparticles on flat alumina substrate. **(a)** Nanoparticles without peptide (PS-COOH). **(b)** Nanoparticles with peptide (PS-COO-(RF)_4_).

The nanoparticles are polystyrene (PS), which were pretreated with carboxyl (COOH) groups on the surfaces. So we could functionalize their surfaces with arginine/phenylalanine peptide ((RF)_4_). This is a positively charged peptide, consisting of alternating sequence of arginine (R) and phenylalanine (F) amino acids, which can be used to fabricate fibers and hydrogel matrices for potential biological applications^21^,^22^. This peptide of (RF)_4_ has high affinity to react with carboxyl groups on the polystyrene nanoparticles (PS-COOH) to form strong crosslinking bonds (PS-COO-(RF)_4_) (Fig. 4a). The functionalized nanoparticles were characterized by Fourier transform infrared (FTIR) spectroscopy as seen in Fig. 4b. Several specific peaks at 633, 1236, and 3435 cm^−1^ reveal the presence of N-H wag, C-N stretch and N-H stretch functional groups on nanoparticles (PS-COO-(RF)_4_), respectively. Moreover, there are strong absorption bands at 1045 and 1706 cm^−1^, which indicate the formation of C-O, C=O, and C=N stretch. So this result shows that the carboxylated nanoparticles are functionalized with the peptide group (RF)_4_ successfully. In this study, influence of the peptide on the size of the nanoparticles can be negligible, but it has significant influence on the surface properties. Figure 5 shows the arrangement of nanoparticles, which have been dispensed on a flat alumina (Al_2_O_3_) substrate in the solution and then dried on the hot plate. The particles without the peptide of (RF)_4_ are arranged in good order, or self-assembled (Fig. 5a), but the particles with (RF)_4_ are placed randomly (Fig. 5b). This difference has been caused by the electric charge of the nanoparticles and that of the alumina surface. The nanoparticles with carboxyl group (COOH) are charged negatively and those with peptide ((RF)_4_) are charged positively, while the surface of alumina is charged positively. When the particles and the substrate have different charge, the particles are arranged as crystal growth, or self-assembled. But when the particles and the substrate have the same charge, the particles are repelled from the surface and maintain their mobility in aqueous solution^15^, which results in the random arrangement of the nanoparticles as seen in Fig. 5b. Since the working template in this study is aluminum oxide also, the nanoparticles with (RF)_4_ will have higher mobility than those without it while they are injected into the pores. This means that the nanoparticles with (RF)_4_ can move more freely with the injected flow, resulting in higher chances for them to be stacked, or embedded, more densely in the pores.

### Hydraulic characteristics of nano sand filter

**Figure 6:**
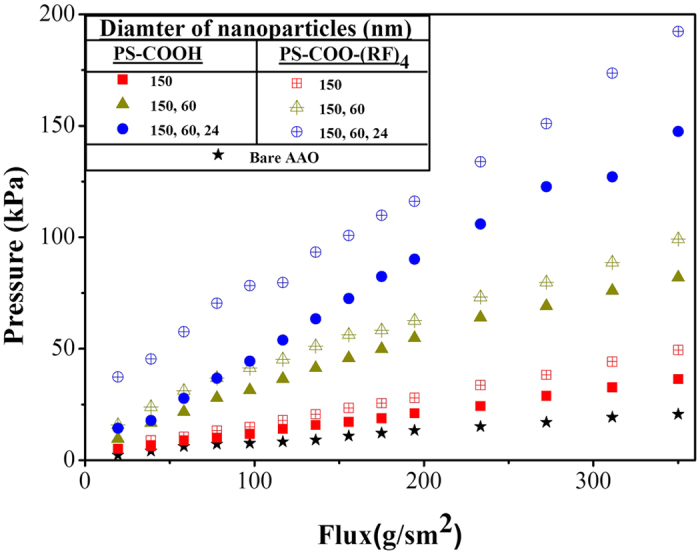
Hydraulic pressure drops across the working templates for various flow fluxes. Differential pressure of water flow was measured to obtain the pressure drop across the AAO template embedded with nanoparticles (Supplementary Fig. 2).

One of the major purposes of this study is to fabricate voids in nanoscale dimension. However, geometry of the voids between the nanoparticles is complicated and they cannot be evaluated on the cross sections. Therefore, we measured hydraulic pressure drop across the nanoparticle-embedded AAO templates so that we could compare the voids relatively for various combinations of the nanoparticles (Supplementary Fig. 2). Deionized (DI) water was pumped with a syringe pump and differential pressure across the AAO template was measured (Romemount 3051TG Transmitter, Jackwell Inc.). Figure 6 shows the hydraulic pressure drop for various fluxes and various combinations of nanoparticles in the AAO templates. As smaller nanoparticles are embedded, the voids between the nanoparticles are expected to be narrower, which means that hydraulic passages become narrower. Accordingly, the results are showing that the AAO templates with smaller nanoparticles have produced higher pressure drop for the same flux than those with larger particles. Therefore, this hydraulic measurement suggests that the voids between the nanoparticles, which are the hydraulic passages, could be modulated by embedding nanoparticles with different sizes. And this result also shows that the nanoparticles are trapped, or confined, stably inside the pores. The hydraulic characteristics vary consistently with the flow flux, so it can be inferred that there was no serious loss of the particles during the tests. For this reason, further treatment for stabilization of the particles was not necessary as long as the flow is applied from the top side of the AAO. And it is also remarkable that the particles with (RF)_4_ produced higher pressure drops than those without it for the same size of the nanoparticles. As discussed in the above paragraph, denser embedment is expected when the particles have the same charge with inner wall of the template. Accordingly, this result is showing that the particles with (RF)_4_ are embedded more densely, making smaller voids than those without it.

### Filtration tests with dye molecules

**Figure 7:**
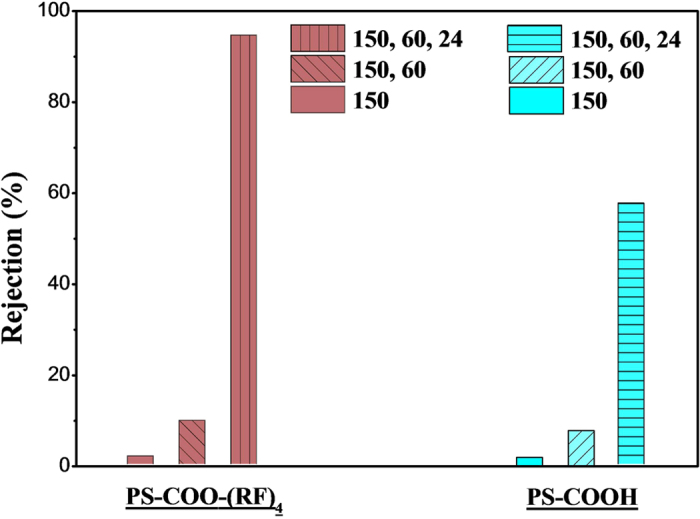
Methylene blue filtration using AAO templates with different combination of nanoparticles. All experiments were performed with flow flux between 21.6~25.5 g/sm^2^.

**Figure 8:**
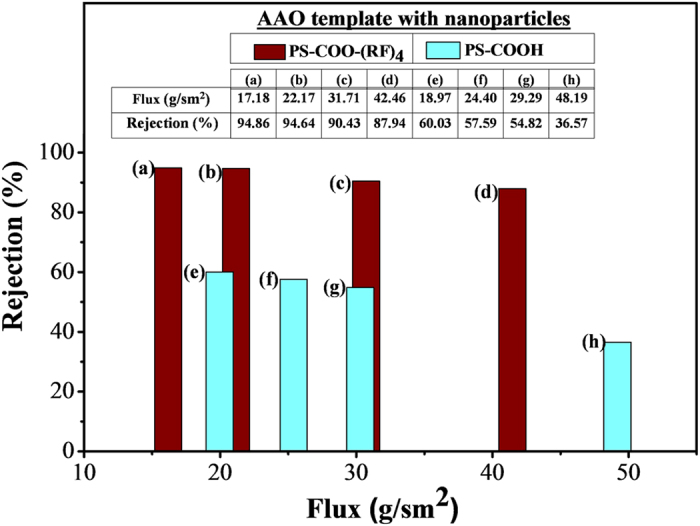
Methylene blue filtration using AAO templates with nanoparticles of 150, 60 and 24 nm. Rejection rates for various fluxes were obtained. The flux through the templates increases from left to right. **(a–d)** AAO templates with nanoparticles with peptide (PS-COO-(RF)_4_). **(e–h)** AAO templates with nanoparticles without peptide (PS-COOH).

**Figure 9:**
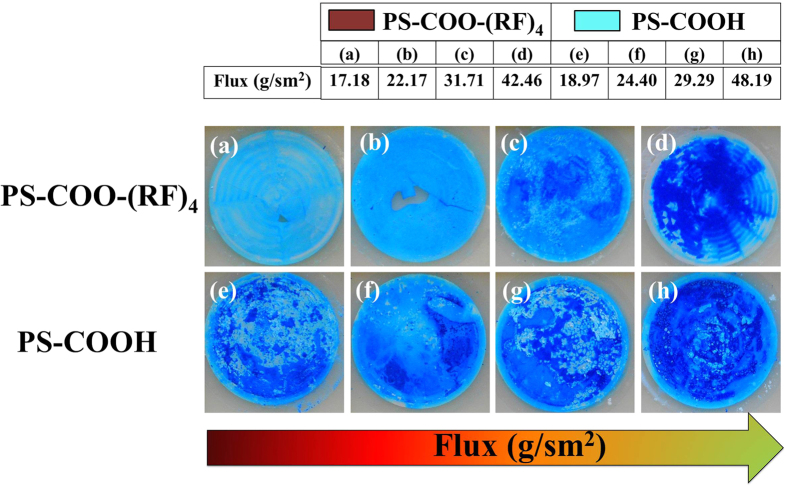
Pictures of the AAO templates after the methylene blue filtration test. **(a–d)** AAO templates with nanoparticles with peptide (PS-COO-(RF)_4_). **(e–h)** AAO templates with nanoparticles without peptide (PS-COOH). The particles of 150, 60 and 24 nm are embedded together for all templates. The flow flux through membrane has been increased from left to right.

Methylene blue is a common dye for biological stain, which is soluble in water and measurable with spectroscopic method^23^,^24^. In this study, this dye was used as an indicator for filtration characteristics because it is charged positively in aqueous solution. And the nanoparticles in this study may have negative charge with carboxyl group, or positive charge with peptide ((RF)_4_). So filtration characteristics have been investigated with the nanoparticle-embedded templates using this dye solution. The nanoparticle-embedded AAO templates were prepared by using the particles of 150, 60 and 24 nm in diameter and they were made into two groups according to the surface functionalization with (RF)_4_ on the particles. The dye solution of 10 mg/L was pressurized in a stirred cell. And the concentration of the solution was measured with ultraviolet (UV)-visible spectroscopy as seen in Supplementary Fig. 3. Firstly, the rejection rates were measured using different combination of nanoparticles as seen in Fig. 7. The rejection rates are quite poor unless the smallest particles (24 nm) were embedded. In other words, the rejection is increased much when all of the particles are embedded together. Furthermore, it is also noticeable that the highest rejections from each group are quite different, which are 94.6% for the particles with peptide and 57.6% for those without it. This result means that the charge of the nanoparticles is effective on filtration performance, as long as the size of voids is comparable to that of the filtration target. Then, further tests were performed only using the templates which have all of the particles (150, 60 and 24 nm) inside. The rejection rate has been obtained for various mass fluxes as seen in Fig. 8. As the flux increases, the rejection decreases slightly for all cases. However, it is evident that the rejection is much higher when the nanoparticles with peptide ((RF)_4_) were used. As discussed in the above paragraph, the templates with peptide-treated particles have produced higher pressure drop, which means that the nanoparticles with peptide are embedded more closely, resulting in smaller voids. So this difference of rejection is partially caused by the embedment, or stacking, of the nanoparticles. However, the rejection is also influenced much by the electric charge of the particles and dye molecules. Since the dye and peptide ((RF)_4_) have the same charge, higher rejection (between 94.8% and 87.9% in Fig. 8a~8d) has been obtained as seen in the result. Meanwhile, the templates without peptide, which have carboxyl group on the nanoparticles instead, have opposite charge to the dye molecules, so the rejection is low and it degrades as the flux increases (between 60.0% and 36.6% in Fig. 8e~8h). These dye tests (Figs 7 and 8) suggest that the void size is critical to the filtration characteristics. So the particle size and its combination need to be selected considering the target. Moreover, it has been also investigated that matching of charges between the target and the nanoparticles can enhance the filtration greatly. For more investigation on the charge-matching issue, the templates have been inspected after the tests as seen in Fig. 9. It shows the templates, which have been disassembled from the stirred cell after experiments and then dried out. Color of the residue is much darker on the templates without the peptide (Fig. 9e~9h), while it is lighter on those with the peptide (Fig. 9a~9d). So these pictures explain that the templates without peptide have produced poor rejection rate because of the negative charge of the nanoparticles and its consequent adsorption of dye onto the nanoparticles.

## Discussion

Nanoparticles have been embedded within the pores of AAO templates in order to fabricate voids in nanoscale dimension. Since the nanoparticles have been confined within the pores, stabilization processes are not required such as cross linking or thermal coalescence. Although the geometry of voids or arrangement of the nanoparticles could not be observed, hydraulic test has shown that the size of voids has been reduced, or could be modulated by using nanoparticles with various sizes. Surfaces of the nanoparticles have been functionalized with arginine/phenylalanine (RF) peptide group, so the filtration or separation in this study is based on charge selection principle, as well as size selection. And experiments have shown that dye molecules could be separated by this way. This study is expected to provide an efficient method for filtration or separation of charged molecules in aqueous solution, as well as a method for fabrication of voids, or pores, in nanoscale dimension. The separation performance strongly depends on the electric charges of the dye and those on the nanoparticles. So future works need to include further investigations for other dye solutions with different charges and various strengths of the charge on the nanoparticles.

## Methods

### Materials

Carboxylated polystyrene nanoparticles with diameter of 150, 60 and 24 nm were purchased from Bangs Lab., Inc. AAO template (Anodisc 25) was purchased from Whatman plc. Methylene blue (M9140) was purchased from Sigma- Aldrich. Nanoparticles were divided into two groups. One group was functionalized with arginine/phenylalanine peptide ((RF)_4_), and other group was used as received. Methylene blue was diluted in DI water to make a solution of 10 mg/L.

### Fabrication

The AAO membranes have asymmetric pores, whose diameter is about 200 nm on the top side and about 100 nm on the bottom side (Fig. 1). Initially, we injected the 150 nm nanoparticle solution into the membrane from the top side using a syringe pump (KDS Legato 210, Thermo Scientific) at the speed of 0.5 ml/min. To avoid formation of additional cake layer on the top surface of the template, we gently rinsed the template with water and then dried it on the hot plate for 60 s at 40 °C. The whole process was repeated to embed further nanoparticles of 60 and 24 nm.

### Characterization

Scanning electron microscopy (SEM) (S-4800, Hitachi) was used to examine the surface morphology of the samples at different experiments condition. FTIR spectra were obtained with Bruker Tensor 27 FT-IR. Romemount 3051TG Transmitter, Jackwell Inc was used to measure the pressure across of DI water across the AAO template. The experiments with methylene blue were performed using a stirred cell (Stirred cell 8010, EMD Millipore AmiconTM). A Shimadzu UV-2450 spectrophotometer was used to measure the concentration of methylene blue solution with the wavelength from 220 to 850 nm.

## Additional Information

**How to cite this article**: Phuong, N.T. *et al*. Nano sand filter with functionalized nanoparticles embedded in anodic aluminum oxide templates. *Sci. Rep.* **6**, 37673; doi: 10.1038/srep37673 (2016).

**Publisher's note:** Springer Nature remains neutral with regard to jurisdictional claims in published maps and institutional affiliations.

## Supplementary Material

Supplementary Information
