# Supplementary material for: Nano sand filter with functionalized nanoparticles embedded in anodic aluminum oxide templates
Source: Sci Rep. 2016 Nov 23;6:37673. doi: 10.1038/srep37673 (PMC5120303; doi:10.1038/srep37673)
Supplement: Supplementary Information [file srep37673-s1.pdf]

# Supplementary Information

**Title: Nano sand filter with functionalized nanoparticles embedded in anodic aluminum oxide templates**

Nguyen Thi Phuong, Anugrah Andisetiawan, Do Van Lam, Jeong Hwan Kim, Doo-Sun Choi, Kyung-hyun Whang, Jeasun Nham, Yoon Jeong Lee, Yeong-Eun Yoo & Jae Sung Yoon

Supplementary Figure 1. Overall view of the cross section of AAO template with nanoparticles.

Supplementary Figure 2. Schematic of test apparatus for hydraulic pressure drop.

Supplementary Figure 3. Relationship between concentration of methylene blue solution and light absorbance and transmittance.

Supplementary Note 1. Relationship between concentration of methylene blue solution and light transmittance.

Supplementary References.

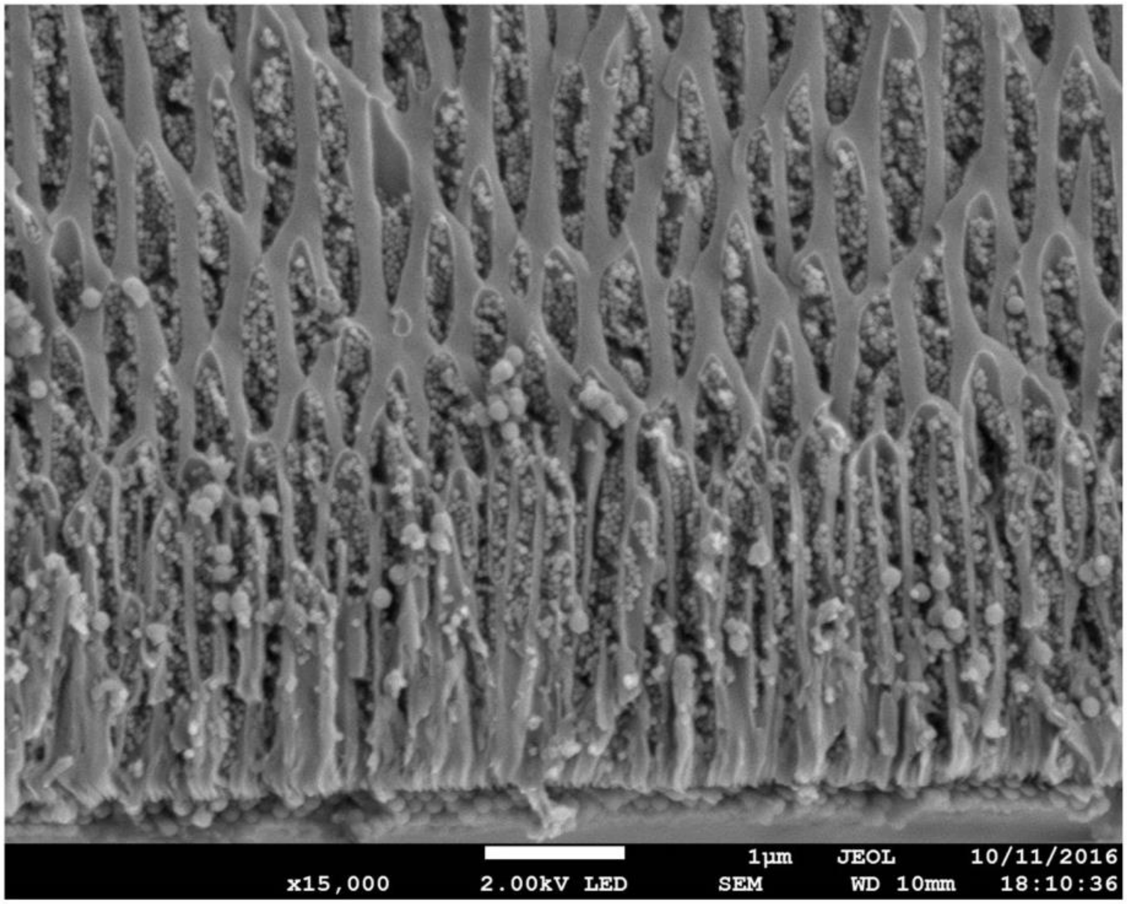


Supplementary Figure 1. Overall view of the cross section of AAO template with nanoparticles. The pores of AAO template are branched, so they are larger on top side and smaller on bottom. The nanoparticles with various sizes are embedded in the pores.

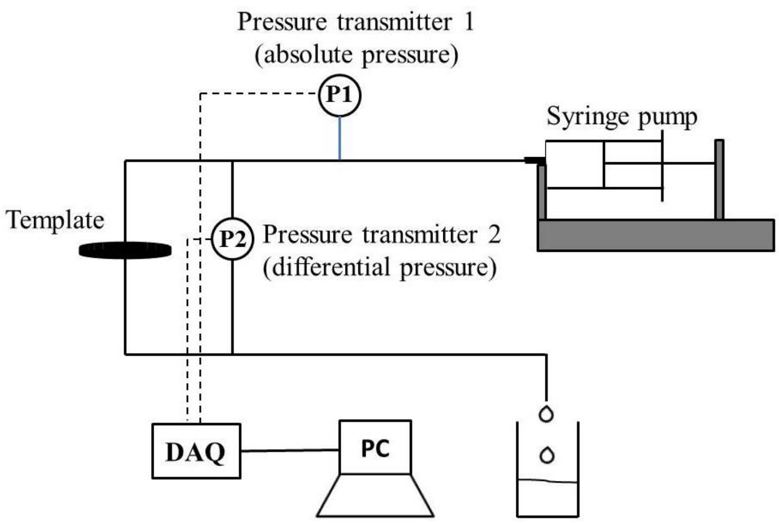


Supplementary Figure 2. Schematic of test apparatus for hydraulic pressure drop. The AAO template with nanoparticles has been put into a membrane holder so that the surface with larger pores faces the water flow. Differential pressure from pressure transmitter 2 was measured to obtain the pressure drop across the template as seen in Fig. 6.

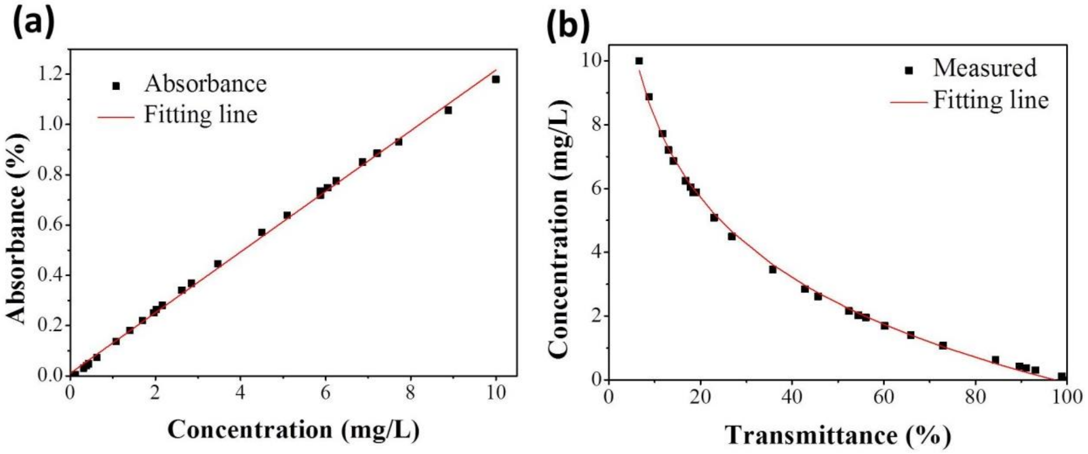


Supplementary Figure 3. Relationship between concentration of methylene blue solution and light absorbance and transmittance. a, Changes of light absorbance for various concentrations of methylene blue solution. b, Correlation of concentration and light transmittance.

Supplementary Note 1. Relationship between concentration of methylene blue solution and light transmittance.

The relationship between absorbance ($A$) and transmittance ($T$) in this study is based on Beer-Lambert law [Supplementary references 1,2] as follows:

$$A = \log_{10}\left(\frac{1}{T}\right) = \log_{10}\left(\frac{100}{T}\right) = 2 - \log T \quad (1)$$

$$A = \varepsilon l C \quad (2)$$

in which $\varepsilon$ is the molar absorptivity, $l$ is the length of dye solution that the light passes through, and $C$ is the concentration of dye solution. The transmittance profile of methylene blue has been measured at the wavelength of 664 nm. The absorbance profile is linearly fitted based on the measured data (Supplementary Fig. 2a):

$$A = 0.01143 + 0.12048C \quad (3)$$

As a result, the relationship between concentration of methylene blue solution and light transmittance has been obtained as follows.

$$C_{fittingline} = \frac{1.9857 - \log T}{0.12048} \quad (4)$$

Supplementary references


1. Zaraska, L., Kurowska, E., Sulka, G. G. & Jaskuta, M. Porous alumina membranes with branched nanopores as templates for fabrication of Y-shaped nanowire arrays. *J Solid State Electrochem* **16**:3611–3619 (2012)
2. Schmid, F. X. Biological macromolecules: UV-visible spectrophotometry. *eLS*, doi: 10.1038/npg.els.0003142 (2001).
3. Behera, S., Ghanty, S., Ahmad, F., Santra, S. & Banerjee, S. UV-visible spectrophotometric method development and validation of assay of paracetamol table formulation. *J. Anal. Bioanal. Techniques* **3,** 1000151 (2012).
